# Assessment of dose reliability in radiotherapy practices in Türkiye: A multicenter study

**DOI:** 10.1002/acm2.70204

**Published:** 2025-08-31

**Authors:** Murat Köylü, Ziya Hazeral, Bülent Yapıcı, Elfide Yılmaz, Nezahat Olacak, Hatice Bilge, Serdar Özkök

**Affiliations:** ^1^ Department of Radiation Oncology Ege University Faculty of Medicine Izmir Türkiye; ^2^ Department of Radiation Oncology Acıbadem University Faculty of Medicine Istanbul Türkiye; ^3^ Occupational Health and Safety Unit Kdz. Ereğli State Hospital Zonguldak Türkiye; ^4^ Istanbul University Institute of Oncology Istanbul Türkiye

**Keywords:** radiotherapy centers, TLD, Türkiye dose audit

## Abstract

**Background:**

Dose accuracy in radiotherapy is crucial for treatment outcomes and patient safety. Ensuring dose accuracy allows for effective targeting of tumors while protecting surrounding healthy tissues. The quality assurance programs implemented worldwide by the International Atomic Energy Agency (IAEA) and the World Health Organization (WHO) are among the most effective methods to enhance dose reliability, particularly in developing countries.

**Purpose:**

This study aims to comprehensively evaluate the reliability of treatment doses applied in radiotherapy centers across Türkiye and to determine the impact of national dose control processes.

**Methods:**

The study, thoroughly analyzes dose accuracy in public, private, and university hospitals across six geographical regions of Türkiye. The study investigates whether the treatment doses administered by these centers comply with internationally accepted limits using the IAEA/WHO thermoluminescent dosimetry (TLD) audit standards. In these audits, the accuracy and consistency of the doses applied by each center were evaluated and compared with international standards.

**Results:**

Among the 34 participating centers, 6% achieved dose deviations within ±1%, while 68% were within the widely accepted ±5% tolerance range. Notably, 26% of the centers exhibited deviations in the ±4%–5% band. Differences in dose accuracy were observed between center types and geographical regions. These results provide a snapshot of national performance and highlight areas for quality improvement. The results indicate that while dose reliability in Türkiye is generally within acceptable limits, deviations in some regions suggest the need for further standardization among centers.

**Conclusions:**

These findings underscore the importance of sustainable dose control processes at the national level and highlight their role in improving the quality of radiotherapy services in Türkiye. The study emphasizes that future dose calibration programs and training will further enhance dose accuracy across the country, contributing to safe and effective treatment for patients.

## 1 INTRODUCTION

Dose accuracy in radiotherapy is a critical factor that directly influences treatment success. Delivering the planned dose to the patient ensures accurate irradiation of the target tissue while preserving healthy tissues. Failure to achieve dose accuracy may reduce treatment efficacy and cause unwanted side effects. Therefore, continuous monitoring and improvement of dose accuracy in radiotherapy centers are of paramount importance. International organizations such as the World Health Organization (WHO) and International Atomic Energy Agency (IAEA) have been conducting various quality assurance programs for an extended period to ensure dose accuracy in radiotherapy centers.^1^, ^2^ These audits primarily rely on thermoluminescent dosimetry (TLD) calibration systems, which precisely measure the doses delivered by radiotherapy devices and compare them with planned doses. The IAEA/WHO TLD postal dose audit program has evaluated over 8000 radiotherapy irradiations worldwide, contributing to maintaining international dose reliability standards.^3^

However, studies evaluating dose accuracy in radiotherapy centers across different geographical regions are needed. Research on dose reliability in radiotherapy applications is limited in both Türkiye and globally, and findings from such studies can contribute to healthcare improvements.^4^, ^5^ Similar studies conducted in Asian countries have revealed regional variations in dose deviations, emphasizing the importance of local calibration programs.^6^ Within this context, this study analyzes the accuracy of treatment doses applied in 34 radiotherapy centers across different geographical regions of Türkiye. This study was designed to assess the accuracy of dose delivery across radiotherapy centers in Türkiye by evaluating how well participating institutions maintained their output within the ±5% tolerance threshold defined by international standards. The study aims to analyze dose accuracy in radiotherapy centers in Türkiye and contribute to quality improvement efforts at both national and international levels.

## 2 MATERIALS AND METHODS

### 2.1 Participants and study design

**FIGURE 1:**
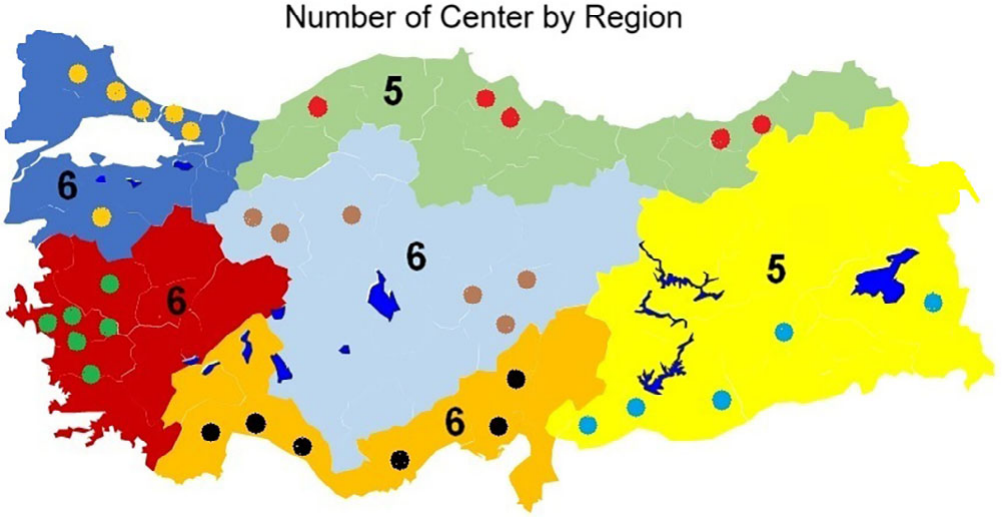
Number of centers by region.

This study was designed as a multicenter analysis encompassing 34 radiotherapy centers in six geographical regions of Türkiye. The participating centers were categorized into three main groups: public, university, and private hospitals. Two public, two private, and two university hospitals were selected from each region. Since the Eastern and Southeastern Anatolia regions were combined as one, the study covered six geographical regions, 20 cities, and 34 centers (Figure 1). Participation in the study was voluntary, and interested centers were provided with information forms and procedural guidelines. Each participating center used its routine clinical treatment planning protocol and dose calculation algorithm to irradiate the phantom under standardized measurement conditions. This approach was intended to reflect each center's actual clinical workflow and enable a realistic comparison of dose delivery accuracy. These centers represent the diversity and dose accuracy of radiotherapy applications in different regions of Türkiye. Although the initial objective was to recruit six centers from each region, participation in the study was based on voluntary consent. Two centers, each from a different geographical region, declined to participate, citing institutional concerns about sharing treatment dose data, which is considered confidential in their departments. Attempts were made to recruit alternative centers from the same regions, but no other institutions volunteered. Therefore, the study was completed with a total of 34 participating centers. The multicenter structure of the study increases the generalizability of the data obtained and provides a comprehensive evaluation of radiotherapy practices throughout Türkiye. All participating centers were provided with a standardized irradiation protocol, a TLD irradiation data form, and a participation form. These documents are included in the supplementary material (Appendices A–C). They ensured uniform implementation of the audit procedure and allowed the collection of essential technical information such as beam type, calibration protocol, and treatment parameters.

### 2.2 TLD calibration and irradiation

In the study, the 6 MV photon energy of the Elekta brand linear accelerator belonging to the Ege University faculty of medicine, radiation oncology department, was used as the reference energy. The reference center used an Elekta Synergy linear accelerator, approximately 5 years old. All irradiations were performed with 6 MV beam energy, and the linac was calibrated prior to each TLD irradiation following the IAEA TRS‐398 protocol. This device was approved to perform within the irradiation criteria by participating in the IAEA/WHO TLD dose comparison program. The limitation of the study is that the doses were compared with the device with 6 MV energy approved by the IAEA instead of directly comparing them with the IAEA laboratory. The reference irradiations were performed at the Ege University Hospital, a nationally recognized radiotherapy center with extensive experience in dosimetry audits. This center has successfully participated in multiple IAEA TLD quality assurance programs. Dose measurements were performed using a method similar to the IAEA/WHO TLD postal dose audit program. The TLDs used in the study were preselected based on their sensitivity, ensuring that the variation in response among them was within 0.1%. This approach allowed the use of a common calibration factor for all TLDs without the need for individual ECC determination. In addition to the irradiation holder, polyethylene capsules for TLDs were designed, producing a total of three holders and 21 capsules. Each center received three TLD capsules, including one irradiated control TLD and two unirradiated TLDs. This study was designed to assess whether treatment units deliver the correct absolute dose under a standard reference geometry, independent of the clinical protocol complexity or treatment planning technique.

The TLDs were irradiated and read at the reference center using a Harshaw 6600 TLD reader. The calibration was performed using a 6 MV linear accelerator that had recently passed an IAEA/WHO TLD audit. Although the calibration was not conducted at an IAEA laboratory, the use of this IAEA‐validated unit provided a traceable and reliable reference. This is acknowledged as a limitation of the study. All participating centers and the reference center used the same photon energy (6 MV), thus minimizing any risk of energy dependence‐related uncertainty.

**FIGURE 2:**
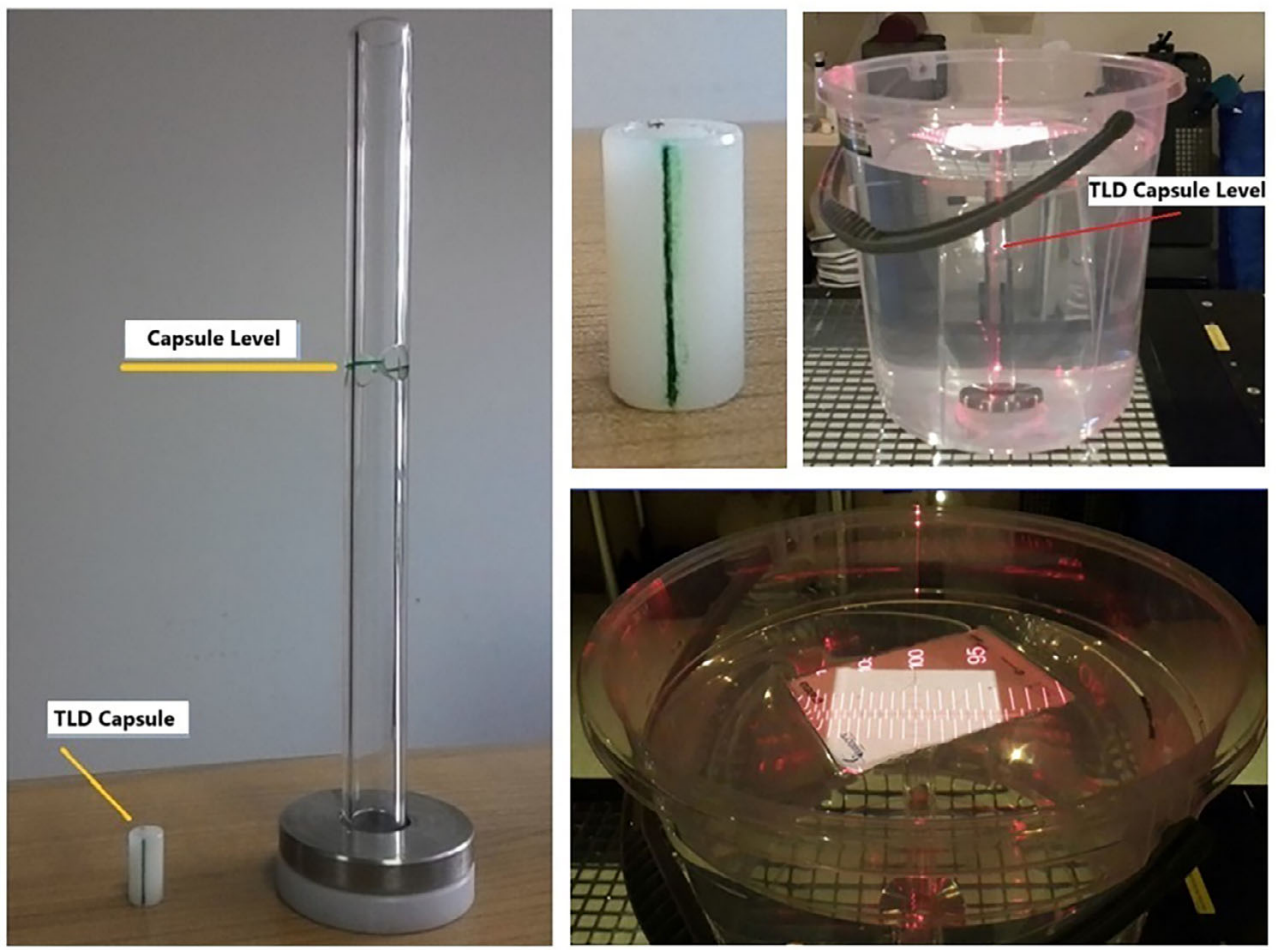
Irradiation apparatus and setup.

TLDs were placed at a 10 cm depth within a 10 × 10 cm irradiation field and at a source‐to‐surface distance (SSD) of 100 cm to ensure a reference dose of 2 Gy (Figure 2). These standardized conditions were chosen to maintain measurement consistency and comparability. Each TLD capsule was positioned inside a custom‐designed water‐equivalent holder placed in a water tank. Reference guide marks on both the capsule and the holder were aligned to ensure proper orientation, with the TLD element placed parallel to the horizontal plane. The capsule was seated at the center of the holder's cavity, and water was added above it to achieve exactly 10 cm depth from the water surface. This setup ensured geometrical precision and accurate depth placement. The couch height was adjusted to obtain 100 cm SSD at the water surface. After irradiating the first capsule, the same setup was used to irradiate the second TLD.

Prior to each irradiation, absolute dose calibration of the reference linac was performed according to the TRS‐398 protocol, and reference measurements were used to evaluate incoming TLDs. Basic mechanical checks (field size, SSD, gantry, and collimator angles) were also conducted to ensure correct beam geometry. These tests are critical for TLDs to make accurate and reliable dose measurements. The IAEA/WHO tolerance limit, defined as the “classical tolerance” value of ±5% in ICRU Report 24, was used as the reference for reliable dose limits.^7^ This limit is used as an international standard to determine acceptable dose deviations in radiotherapy applications. The WHO/IAEA TLD audit programs typically operate with a dose measurement uncertainty of approximately 1.3% (at one sigma level) and use a ±5% tolerance limit as the criterion for acceptable deviation in clinical audits.^7^, ^8^ The same standard was adopted as the reference criterion in this study.

To evaluate dose accuracy, absolute deviation percentages were calculated, and distributions were analyzed based on geographical region and center type. Furthermore, these results were compared with data from similar studies in the literature to assess their alignment with international standards. Such comparisons are crucial for evaluating whether radiotherapy practices in Türkiye comply with global benchmarks.

## 3 RESULTS

### 3.1 Nationwide dose assessment

**FIGURE 3:**
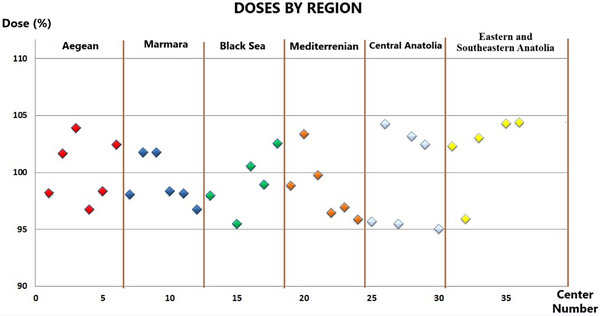
Center doses by region.

Among the 34 participating centers, 2 centers (5.9%) had dose deviations within ±1%, 9 centers (26.5%) within ±1%–2%, 5 centers (14.7%) within ±2%–3%, 8 centers (23.5%) within ±3%–4%, and 10 centers (29.4%) within ±4%–5%. No center exceeded the ±5% tolerance threshold. The highest positive deviation was recorded as +4.43%, while the highest negative deviation was –4.92%. The average dose deviation across all centers was calculated as 2.79%, with a standard deviation of 1.27%. These results indicate that the overall dose delivery reliability of radiotherapy centers in Türkiye is within acceptable limits. The regional distribution of dose deviations is presented in Figure 3.

### 3.2 Dose assessment by center type

**FIGURE 4:**
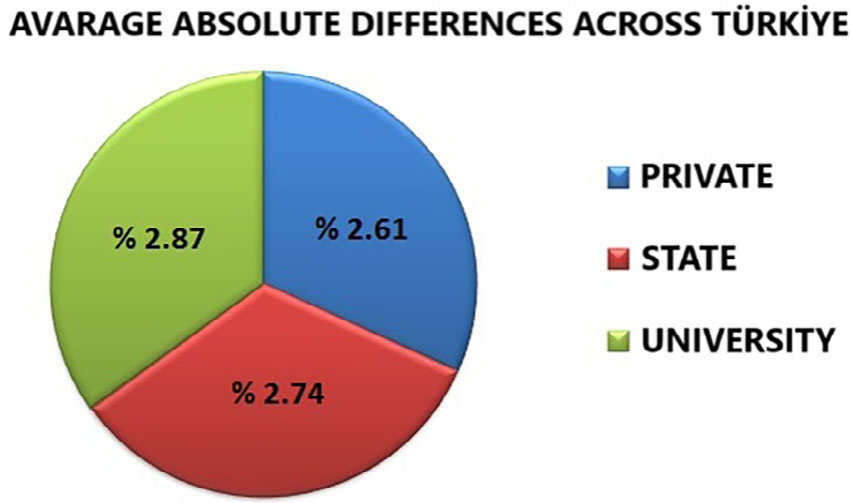
Average absolute dose differences across Türkiye by center type.

When analyzing absolute dose deviations by center type, private hospitals exhibited the lowest dose deviations, whereas university hospitals had the highest deviations (Figure 4). In the analysis by center type, the average absolute dose deviation was calculated as 2.61% for private hospitals, 2.74% for state hospitals, and 2.87% for university hospitals. All center types remained well within the ±5% tolerance threshold. When further broken down by geographic region, university hospitals exhibited relatively higher deviations in some areas (e.g., 4.4% in Southeastern Anatolia and 3.7% in Central Anatolia), while private centers demonstrated the lowest deviations in the Black Sea (1.0%) and Marmara (1.9%) regions. These findings suggest that dose delivery performance may vary depending on institutional type and regional differences in equipment quality, calibration protocols, and staff experience.

### 3.3 Number of centers in the dose acceptance range

**FIGURE 5:**
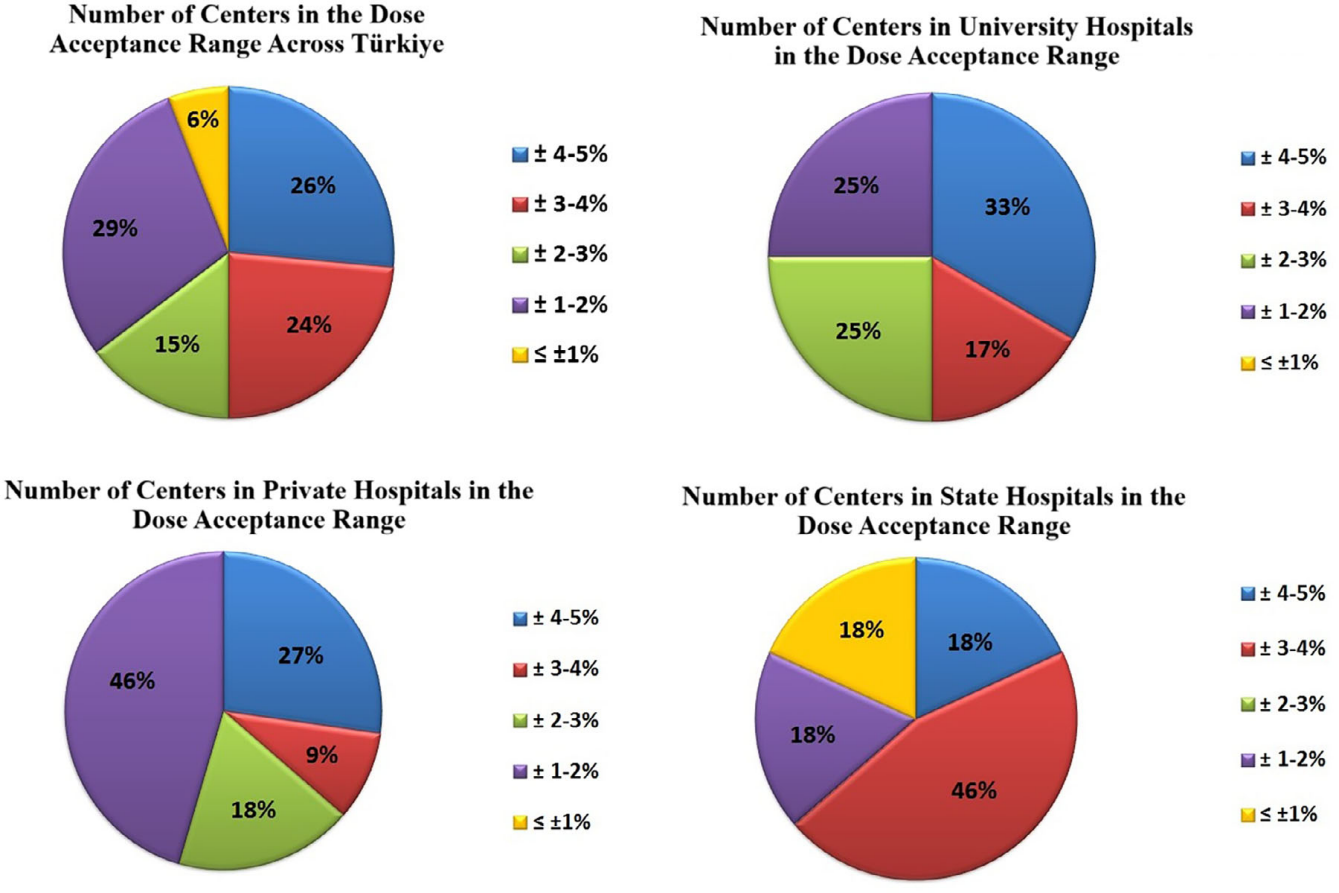
Number of centers in the dose acceptance range.

When evaluating the number of centers within the dose acceptance range across Türkiye, only 6% of centers were found to have dose deviations within ±1%, while 26% fell within the ±4%–5% range. These results indicate that many centers are operating near the upper limit of the internationally accepted ±5% threshold. When assessed by center type, none of the university or private hospitals achieved deviations within ±1%. In contrast, the proportion of centers with deviations in the ±4%–5% range was 33% for university hospitals, 27% for private hospitals, and 18% for public hospitals. This distribution suggests that dose accuracy is more likely to approach the tolerance limit in university hospitals, highlighting the need for strengthened quality assurance practices in these institutions. All relevant data are presented in detail in Figure 5.

### 3.4 Regional dose assessment

In regional analyses conducted independently of the center type, it was determined that deviations were lowest in the Black Sea region and highest in the Central Anatolia region. Regional analysis of absolute dose deviations revealed notable differences among Türkiye's six geographical regions. The lowest average deviation was observed in the Black Sea region (1.8%), followed by Marmara (2.0%) and Aegean (2.5%). In contrast, Central Anatolia (3.9%) and Eastern & Southeastern Anatolia (3.6%) had the highest regional averages, indicating relatively larger inconsistencies in dose delivery.

**FIGURE 6:**
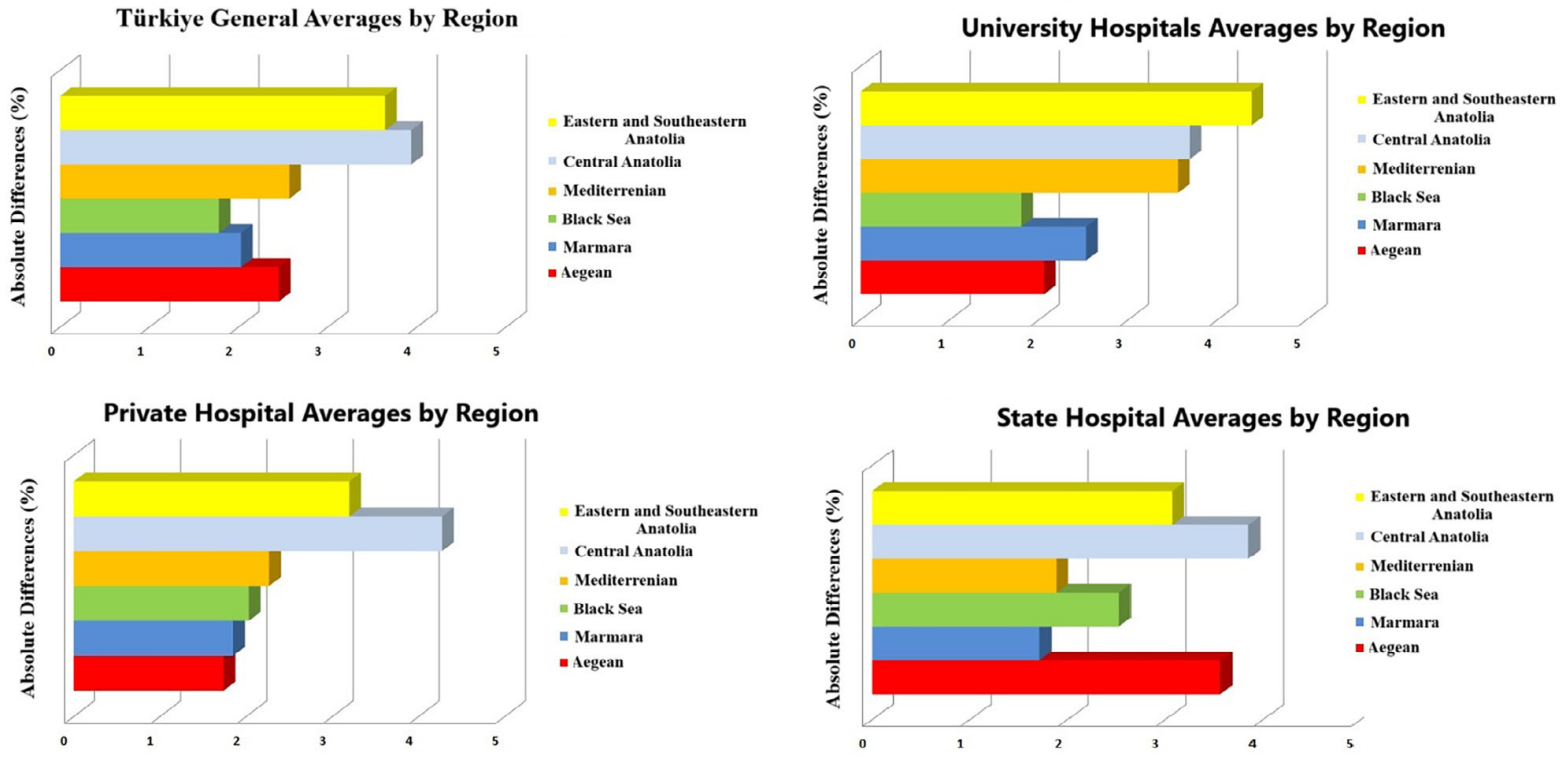
Absolute dose differences by region.

When further stratified by institution type, university hospitals exhibited the highest average deviations in most regions—4.4% in the East and Southeast and 3.7% in Central Anatolia—whereas private hospitals generally showed more consistent performance, particularly in the Black Sea (1.0%) and Marmara (1.9%) regions. These findings suggest that regional disparities in staffing, calibration protocols, and equipment infrastructure may contribute to variations in dose delivery accuracy across the country. Related visual comparisons are presented in Figure 6.

### 3.5 Professional experience and dose deviation

**FIGURE 7:**
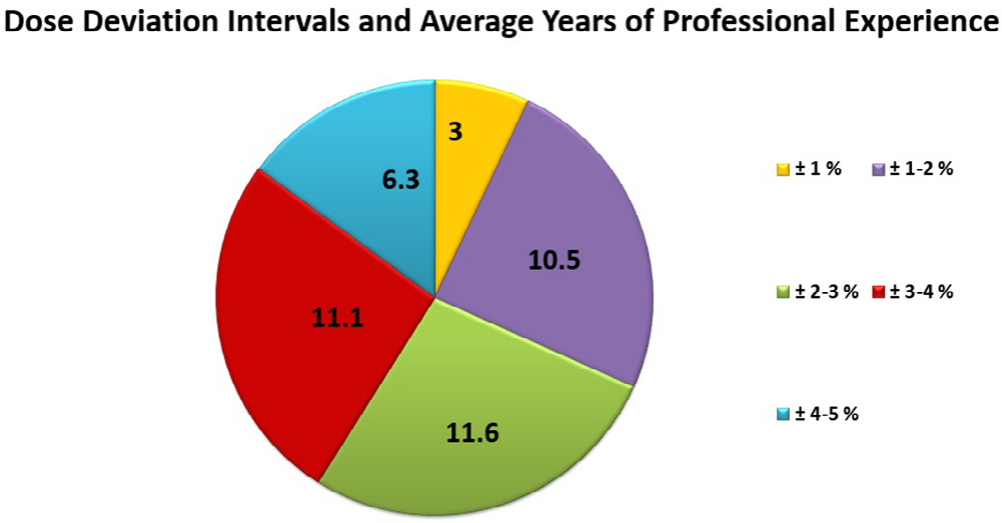
Relationship between dose deviation intervals and the average years of professional experience across participating centers.

To evaluate the potential impact of professional experience on dose accuracy, average years of experience were analyzed by region and institution type. The Marmara and Mediterranean regions had the highest average experience, with 11 and 12 years, respectively, while the Black Sea region had the lowest at 7 years. Among institution types, university hospitals reported the highest average experience (12.7 years), followed by private centers (8.2 years) and state hospitals (5.8 years). Interestingly, there was no direct correlation between experience and dose accuracy. Centers with deviations within ±1% had an average experience of only 3 years, while those in the ±2%–3% and ±3%–4% ranges had higher average experience levels of 11.6 and 11.1 years, respectively. These findings suggest that dose accuracy may be influenced not only by professional experience but also by factors such as protocol implementation, equipment calibration routines, and the local quality assurance culture. The distribution of professional experience by dose deviation intervals is illustrated in Figure 7.

## 4 DISCUSSION

The findings of this study provide a comprehensive evaluation of dose reliability in radiotherapy centers across Türkiye. Accordingly, dose reliability in radiotherapy centers in Turkey is generally within acceptable limits. However, according to the findings, it was also determined that dose deviations varied regionally, by center type, and throughout Türkiye. It has been observed that dose deviations are higher in some geographical regions and center types. The observed differences highlight the need for standardization and more stringent quality assurance protocols across center types and geographic regions.

The primary aim of this study was to evaluate the absolute dose accuracy of radiotherapy centers across Türkiye under a standardized and simplified geometry. Rather than analyzing the entire clinical workflow, this approach was chosen to provide an objective and comparable indicator of treatment delivery performance. It is important to note that the delivery of safe and effective radiotherapy encompasses a much broader process, including target delineation, imaging, planning, treatment execution, and follow‐up. Therefore, the findings of this study should be interpreted specifically within the scope of dose accuracy. This limitation was intentionally defined at the outset, in alignment with international dosimetric audit methodologies.

### 4.1 Dose assessment by region

In our study, the doses of centers across Türkiye were examined on a regional basis. While the Black Sea Region is notable for its low dose deviations, the Central Anatolia Region shows the highest dose deviations. Several factors could contribute to these regional disparities, including differences in equipment age, calibration frequency, and staff training levels. It is thought that the high dose deviations, especially in the Central Anatolia Region, indicate that there may be deficiencies in the quality control practices of some centers in this region. These findings show that regional differences have a significant effect on dose accuracy.

In this study, the observed dose deviations across different geographical regions of Türkiye are not merely numerical differences but may reflect underlying structural imbalances in the national healthcare delivery system. One of the primary motivations for regional comparison is the tendency of experienced medical physicists to seek employment in larger, more developed cities. As a result, differences in institutional resources and staffing profiles across regions may have affected the consistency of quality assurance procedures, although this could not be directly assessed in this study.

Additionally, although the government makes substantial efforts to distribute advanced radiotherapy equipment throughout the country, devices in more densely populated western and central regions are used more frequently, undergo more regular maintenance, and are generally of a newer technological generation. These disparities may contribute to regional differences in the consistency and accuracy of dose delivery. While this study does not include direct socioeconomic data, such regional comparisons may serve as observational proxies for broader systemic variables such as staff qualifications, equipment quality, and clinical workload. These considerations have now been clarified in the revised discussion, with explicit acknowledgment that causality cannot be conclusively established based on the current dataset.

Similarly, Liu et al.^9^ stated that regional differences are an important factor affecting dose deviations in their studies conducted in Asian countries. In their studies, they stated that lower dose deviations were observed, especially in certain regions, and that this was related to regional quality control practices and calibration of the devices used. Our findings in Turkey indicate that regional dose calibration programs should be strengthened.

In this study, the concept of “continuous improvement” does not refer to surpassing the inherent physical limitations of dosimetric systems, but rather to enhancing clinical practice through structured feedback, learning, and targeted education. Following the audit, focused calibration training sessions were organized in regions where significant dose deviations were observed, with the aim of supporting continuous improvement. These practical initiatives are expected to contribute to long‐term enhancement of radiotherapy quality assurance at the national level.

### 4.2 Evaluation of doses by center type

One of the most notable findings is the variation in dose deviations among different hospital types. Analysis by center type shows that dose deviations are lowest in private hospitals and highest in university hospitals. This difference may be due to the fact that private hospitals generally have newer technology, more stringent quality control processes, and regular maintenance programs. It is thought that the higher dose deviations in university hospitals may be related to more complex treatment protocols, higher patient loads, and the use of older equipment in some institutions. It should also be taken into account that dose deviations may be higher due to the intensity of education and research activities. This situation shows that quality control processes need to be improved further in university hospitals compared to public and private hospitals. These findings are consistent with previous studies conducted in other countries where dose accuracy has been shown to be affected by institutional and economic factors.^10^, ^11^ Thwaites^12^ suggested that in university hospitals with more advanced treatment modalities and high clinical workloads, resource and time constraints may occasionally challenge QA consistency, potentially leading to increased dose deviations. This view is consistent with the high deviations seen in the university hospitals in our study. Additionally, Kumar et al.^4^ revealed that differences in quality control processes in private and public hospitals have a significant impact on dose accuracy.

### 4.3 Number of centers in the dose acceptance range

The average dose deviation obtained throughout Türkiye was calculated as 2.79% and the standard deviation as 1.27%. This result shows that the dose reliability in Turkey is compatible with international standards and is generally within acceptable limits. When the number of centers in the dose acceptance range across Turkey is examined, 6% of the centers have doses that differ by less than ±1%, while 26% have dose deviations of ±4%–5%. This shows that some centers do not fully comply with the internationally accepted tolerance limits. In particular, the proportion of centers where dose deviations are between ±4%–5% indicates that quality control processes need to be strengthened. Izewska^13^ emphasizes that TLD audits carried out worldwide should reduce deviations in dose accuracy between centers at the international level. International institutions such as the IAEA^7^ and Thwaites^2^ recommend continuous training programs and quality audits to increase dose reliability. These findings can be considered as an important step toward improving the quality of radiotherapy services in Türkiye. They reveal that more training and audit programs are needed to increase dose accuracy, especially in centers with high dose deviations. In addition, Hossain et al.^5^ stated that dose deviations are larger in radiotherapy centers in developing countries, but local audits and trainings play an important role in reducing these deviations. The IAEA^7^ emphasizes that dose deviations are generally below 3% in centers worldwide and that this value is an internationally accepted target. Although the average dose deviation in Türkiye is in line with this target, it is understood that more quality control measures should be taken to reduce deviations in some centers.

### 4.4 The role of professional experience in inter‐center dose variability

The findings of this study suggest that inter‐center dose variability cannot be solely attributed to the years of professional experience of the medical physicists. In some centers with deviations within ±1%, the average experience was relatively low, while other centers with deviations in the ±3%–4% range had experience levels exceeding 11 years. This indicates that experience alone may not be the primary determinant of dose accuracy; rather, structural and procedural factors may also play critical roles.

These results are consistent with the findings of **Alvarez et al**.^8^, who reported that in large‐scale TLD audit programs, factors such as equipment calibration, user habits, and quality assurance practices were stronger predictors of dose deviation than individual experience. Our study reinforces this view by highlighting that while experience contributes to practice quality, standardized calibration protocols, QA implementation, and institutional consistency may have a greater influence on dose delivery reliability.

### 4.5 Comparison with literature

Similar studies in the literature also show that analyses of dose accuracy in radiotherapy centers in different countries are parallel to the findings obtained in Türkiye. For example, in a study conducted in Poland,^14^ it was stated that treatment doses showed a deviation of 3.5% and that these deviations varied due to differences between centers. Another study conducted in India emphasized that dose deviations in radiotherapy centers were around 3.0% and that higher deviations were observed in some regions.^4^ These findings indicate that radiotherapy practices in India face similar difficulties as practices in Türkiye. When compared to the acceptance limits determined by the IAEA/WHO, it was observed that dose deviations throughout Türkiye remained within the limits of 5%. This shows that radiotherapy centers in Türkiye comply with international standards and generally provide acceptable dose accuracy. For example, in a study in Bangladesh,^5^ it was stated that dose measurements made in radiotherapy centers remained within internationally accepted limits with a deviation of around 4.2%. These findings reveal that radiotherapy practices in Türkiye are in line with international standards and that quality control processes are generally effective.

## 5 CONCLUSION

This study has shown that dose accuracy is generally within acceptable limits in radiotherapy centers throughout Türkiye. However, dose deviations were determined to be higher in some geographical regions and center types. These findings indicate areas where dose quality needs to be improved. It was observed that dose deviations were lowest in private hospitals and highest in university hospitals. This suggests that private hospitals may have stricter quality control processes or may be using newer and more advanced technology. It should be noted that dose deviations may be higher in university hospitals due to the intensity of education and research activities. This situation indicates that quality control processes in university hospitals need to be improved.

In regional analyses, it was determined that deviations were lowest in the Black Sea region and highest in the Central Anatolia region. Low deviations in the Black Sea region indicate that centers in this region may be using better calibrated devices or have stricter quality control processes. High deviations in the Central Anatolia region indicate that centers in this region may need improvements in device calibrations or quality control processes. These findings indicate that regional differences have a significant impact on dose accuracy.

The findings of this study provide important clues for improving dose accuracy in radiotherapy centers throughout Türkiye. It has been revealed that quality control processes need to be improved, especially in university hospitals and the Central Anatolia region. These improvements will increase the quality of radiotherapy services throughout Türkiye and make them compatible with international standards. These findings underline the importance of ongoing training programs, standardized calibration procedures, and increased participation in international quality assurance initiatives. Future studies should focus on long‐term improvements in dose reliability, particularly in regions and institutions where deviations are more pronounced. Dose‐calibration programs and trainings to be implemented in the future will further increase dose accuracy throughout Türkiye and contribute to safe and effective treatment for patients.

The data obtained in this study indicate that inter‐center dose variations cannot be explained solely by the professional experience of medical physicists. The presence of ±3%–4% deviations even in centers with high experience levels suggests that factors such as equipment calibration practices, institutional QA culture, and protocol standardization may play a more decisive role in dose accuracy. These findings highlight the need for audit processes to focus not only on staff experience but also on systematic quality assurance mechanisms.

## AUTHOR CONTRIBUTIONS

Murat Köylü: Conceptualization of the study, study design, data analysis, manuscript writing, and final revision. Development of methodology, statistical review, and scientific interpretation. Yusuf Ziya Hazeral: Coordination of multicenter data collection, preliminary analyses, and preparation of data tables. Bülent Yapıcı: Clinical data evaluation, standardization of radiotherapy procedures, inter‐center harmonization, and data verification. Elfide Yılmaz: Management of data entry processes, literature review, and scientific content validation. Nezahat Olacak: General supervision of the project. Hatice Bilge Becerir: Editorial and language editing. Serdar Özkök: Oversight of the scientific integrity of the study and critical review. All authors have read and approved the final version of the manuscript.

## CONFLICT OF INTEREST STATEMENT

The authors declare no conflict of interest.

## FUNDING

No specific funding was received for conducting the research.

## ETHICS STATEMENT

This study did not involve any human participants, identifiable data, or clinical interventions. Therefore, ethical approval was not required. All data collected were obtained from anonymized quality assurance procedures conducted on standard phantoms.

## Supporting information



Supporting Information

## Data Availability

The datasets generated and analyzed during the current study are available from the corresponding author upon reasonable request. However, to preserve institutional confidentiality, center‐specific or identifying information will not be shared. Anonymized data related to study findings can be made available upon request.
