# Supplementary material for: Assessment of dose reliability in radiotherapy practices in Türkiye: A multicenter study
Source: J Appl Clin Med Phys. 2025 Aug 31;26(9):e70204. doi: 10.1002/acm2.70204 (PMC12398952; doi:10.1002/acm2.70204)
Supplement: Supplementary file 1 — Supporting Information [file ACM2-26-e70204-s001.zip › Appendix C - TLD Irradiation Protocol Sent to Participating Centers.docx]

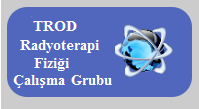
“**Assessment of Dose Reliability in Radiotherapy Practices in Türkiye: A Multicenter Study”**

**TLD Irradiation Protocol**

1. **Calculation of irradiation time:** Using your treatment planning system, calculate the irradiation time required to deliver 200 cGy at a depth of 10 cm with a 10×10 cm² field size and 100 cm SSD.


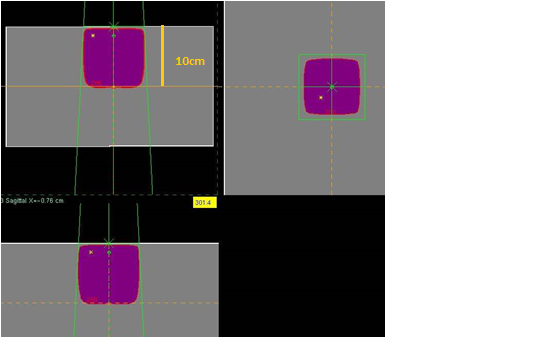


1. **Calibrate your linear accelerator on the day of irradiation:** To minimize the margin of error, if possible, calibrate your linear accelerator on the day of irradiation before irradiating the TLDs.
2. **Do Not Irradiate the Control TLD:** Of the 3 TLDs we have sent, one is designated as a control TLD to eliminate the effect of potential radiation exposure during transport. This TLD must not be irradiated under any circumstances.
3. **Irradiation of Dose TLDs:** Individually irradiate 2 of the 3 TLDs we have sent, delivering 200 cGy to each TLD using the treatment time (MU) calculated with your treatment planning system (TPS).
   1. Place a container that can be filled with water on the treatment couch.


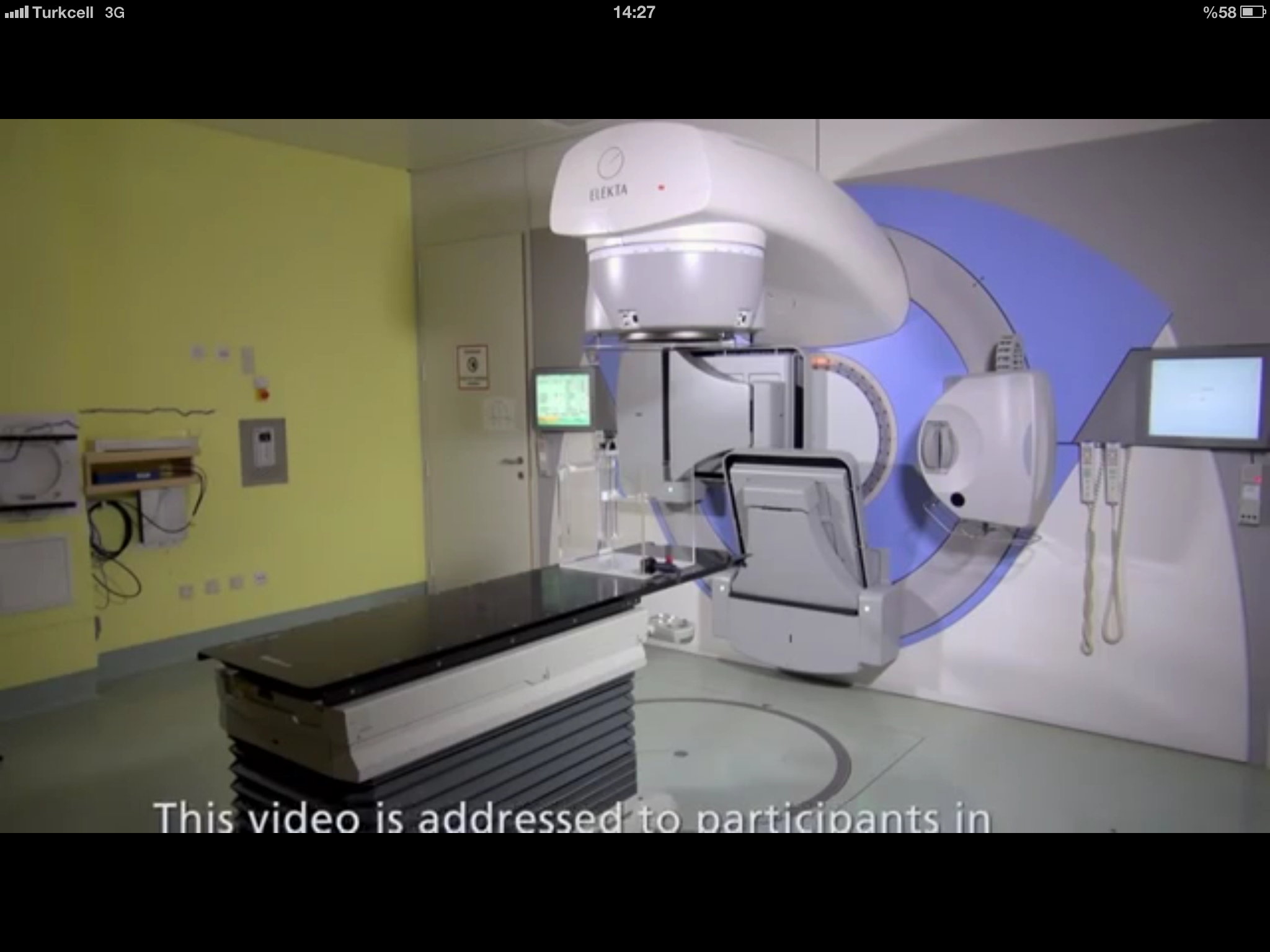


- 1. Place the measurement stick we have sent into the container.


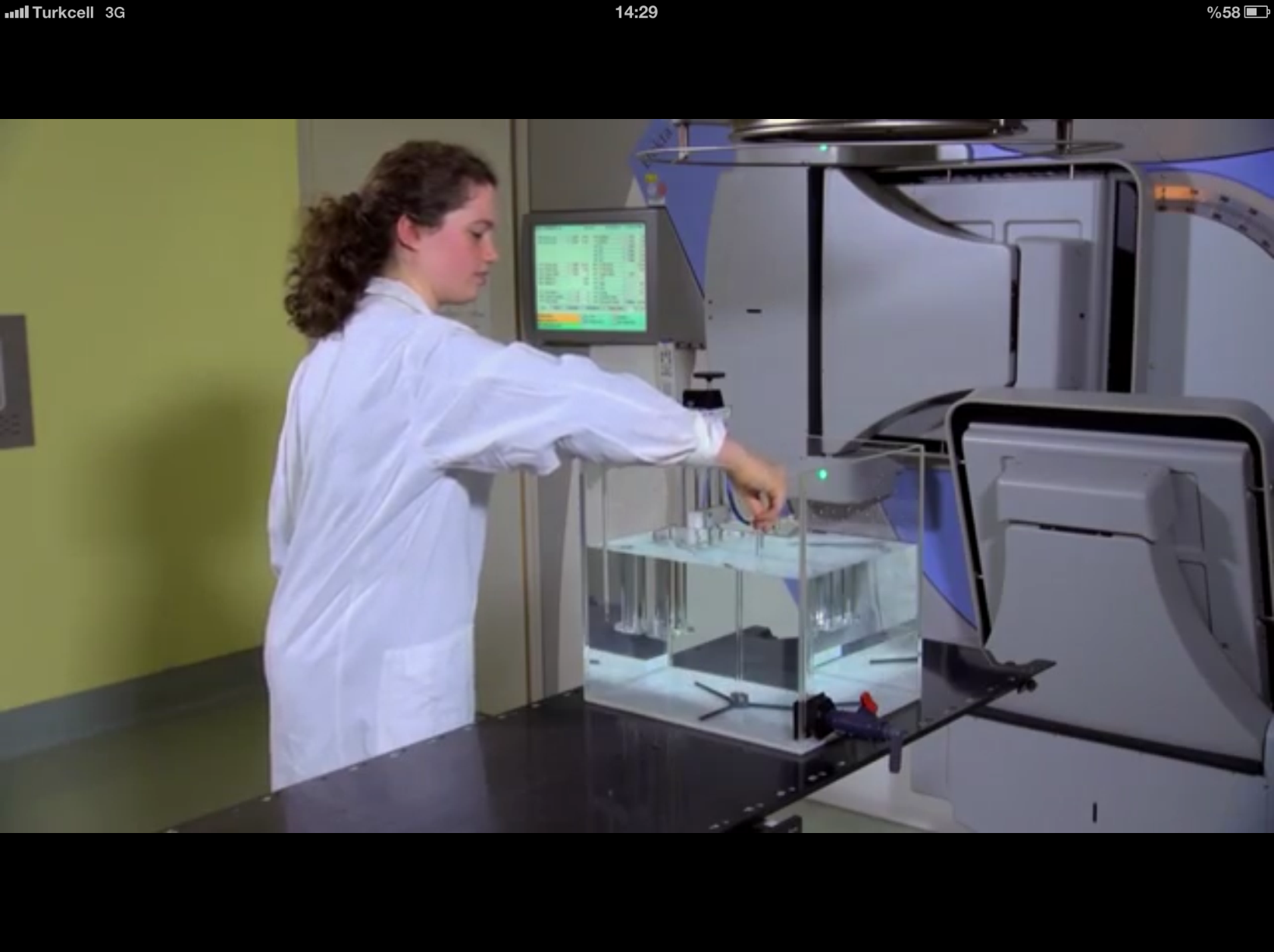


- 1. Fill the container with water up to the level of the cavity where the TLD capsule will be placed.


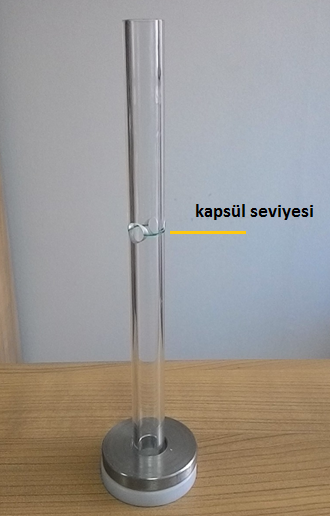


- 1. Place one of the two TLD capsules into the cavity on the measurement stick.**(** **NOTE: While placing the TLD capsule into the cavity, rotate it so that the green alignment line on the TLD capsule is aligned with the green reference line parallel to the center of the cavity on the stick. Make sure that the guide lines on both the stick and the TLD capsule are properly aligned.)**


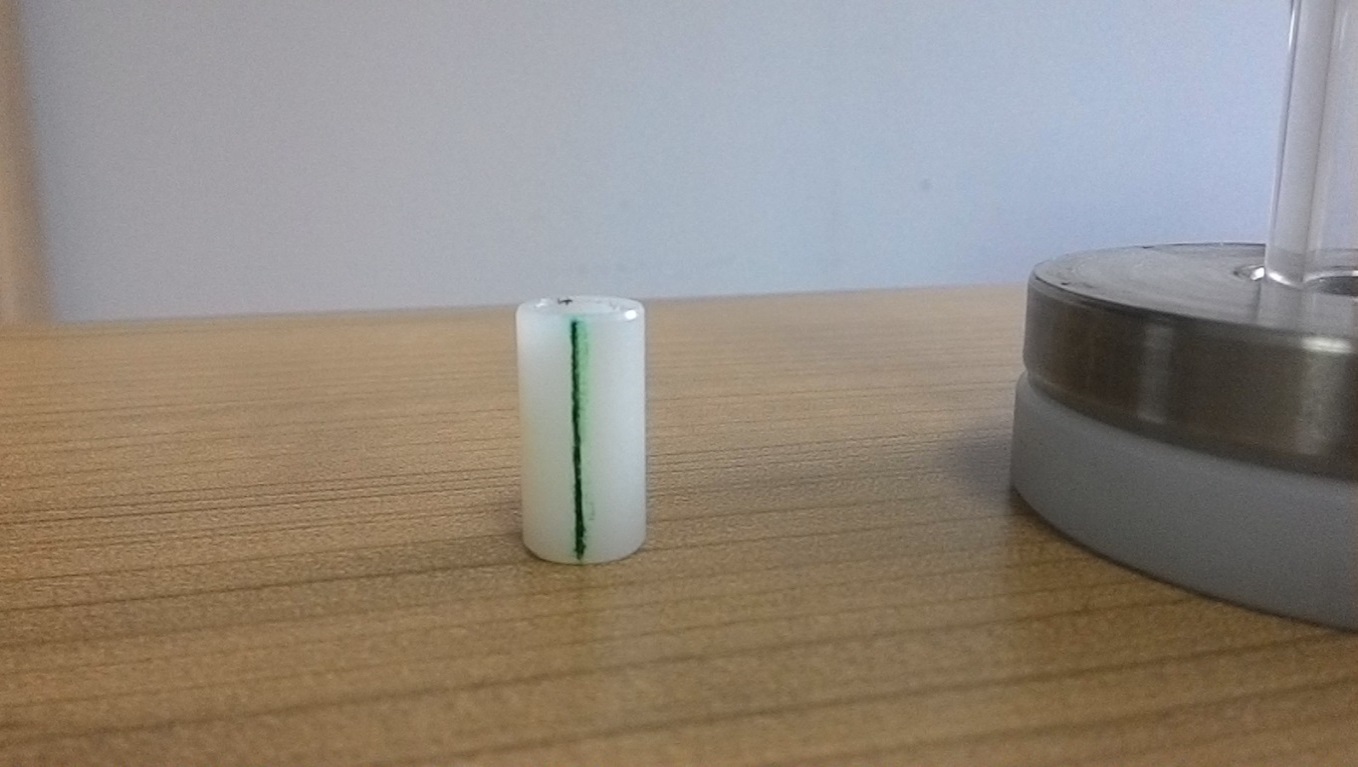


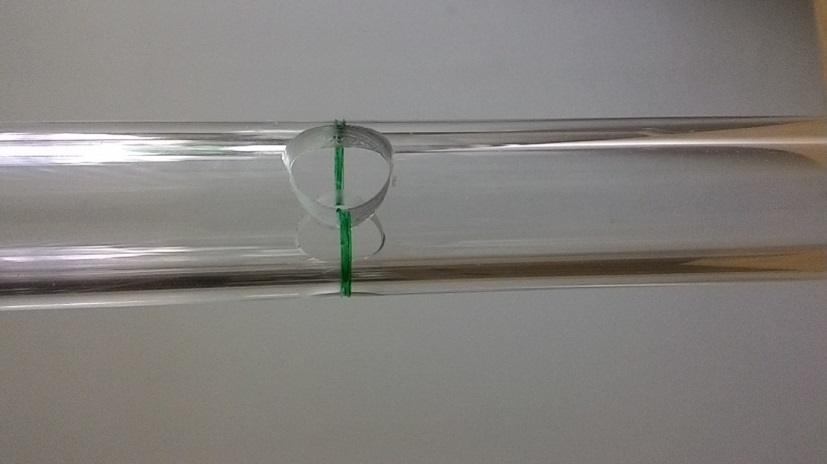


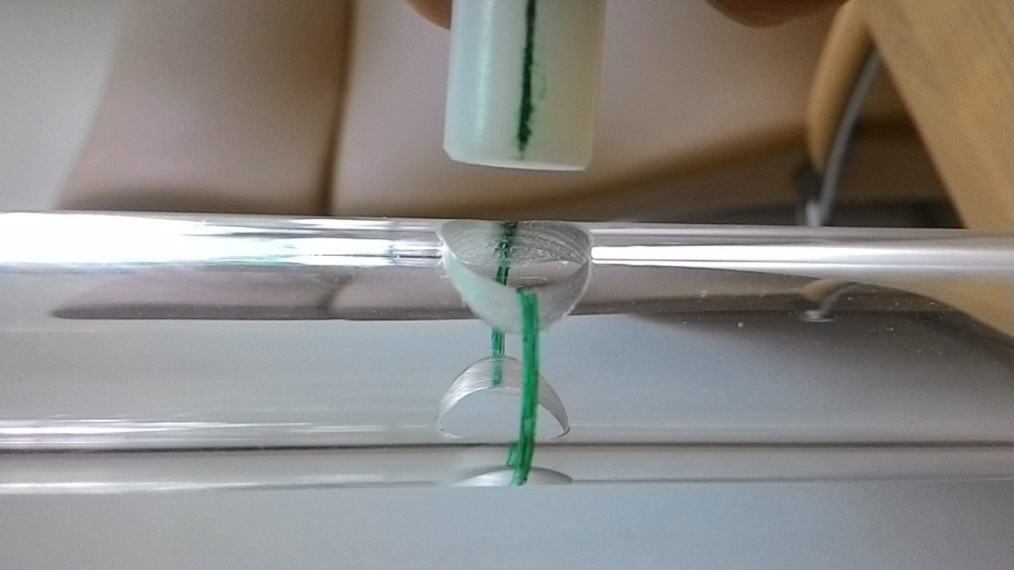

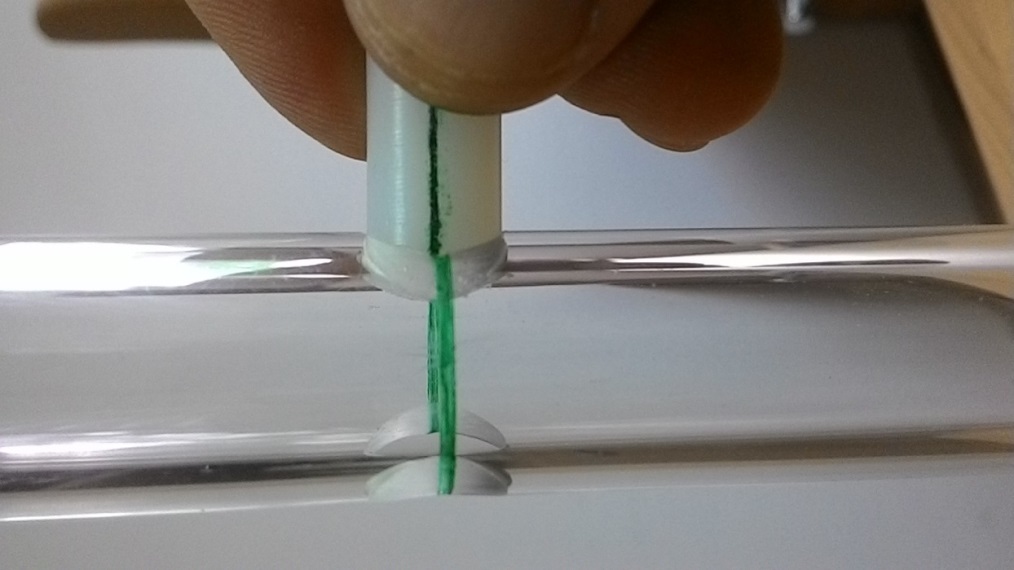


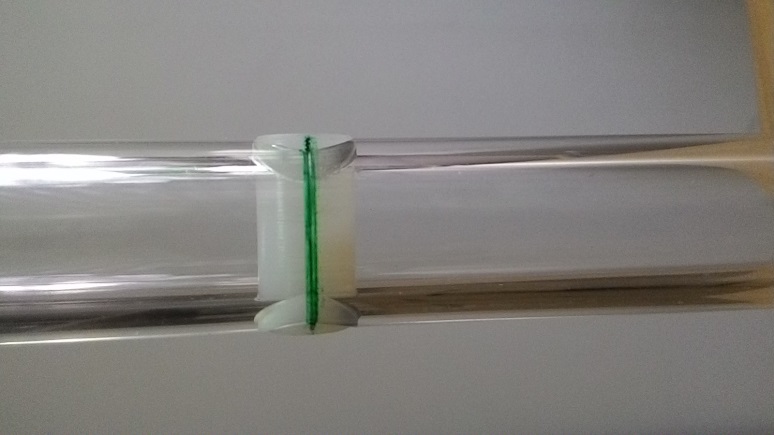

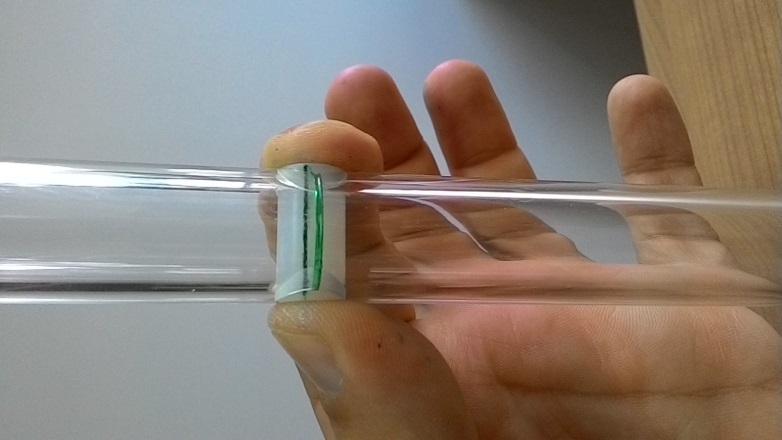

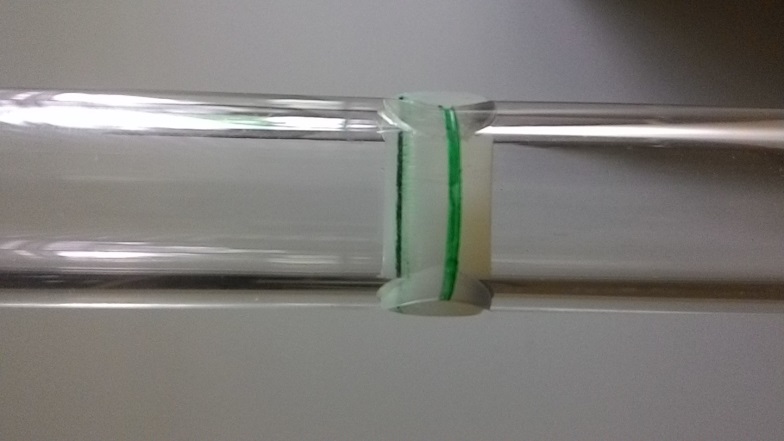


- 1. Place the stick into the container in such a way that it is centered along the central axis of the irradiation field.


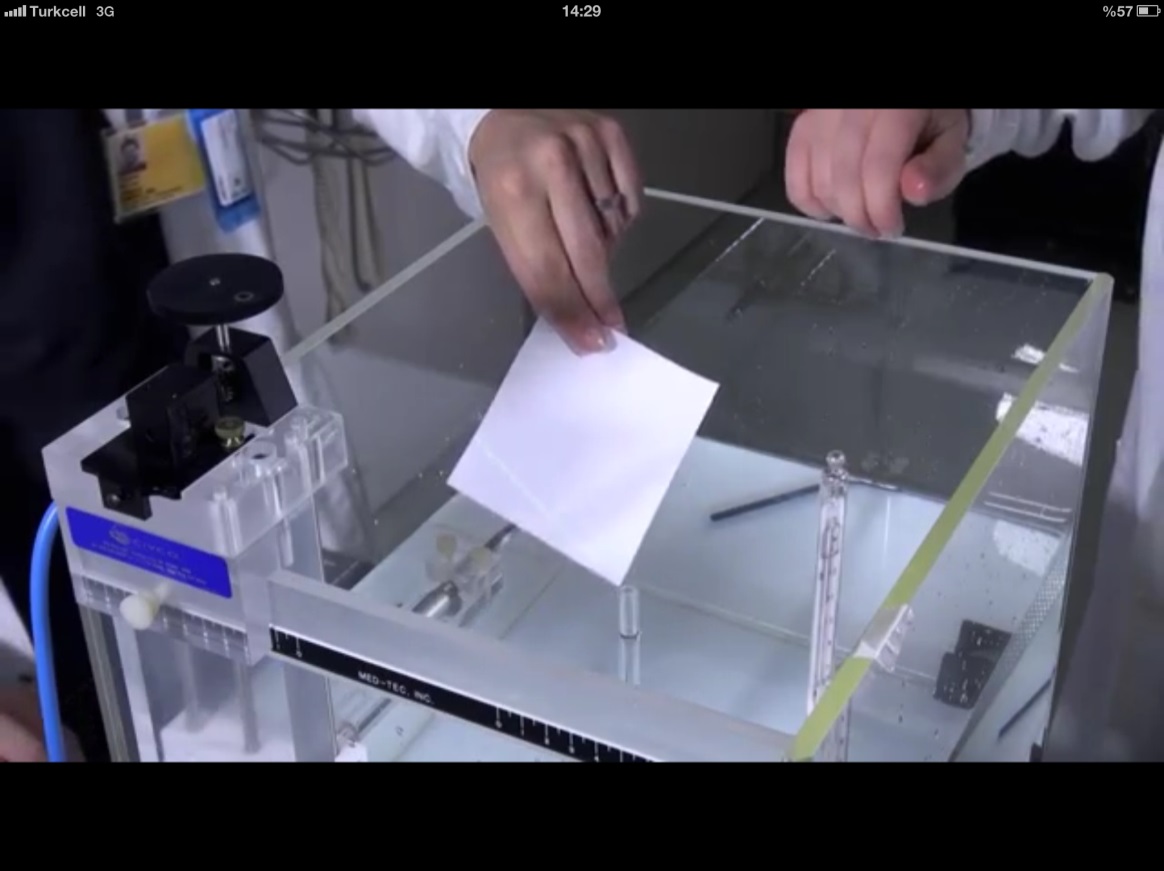


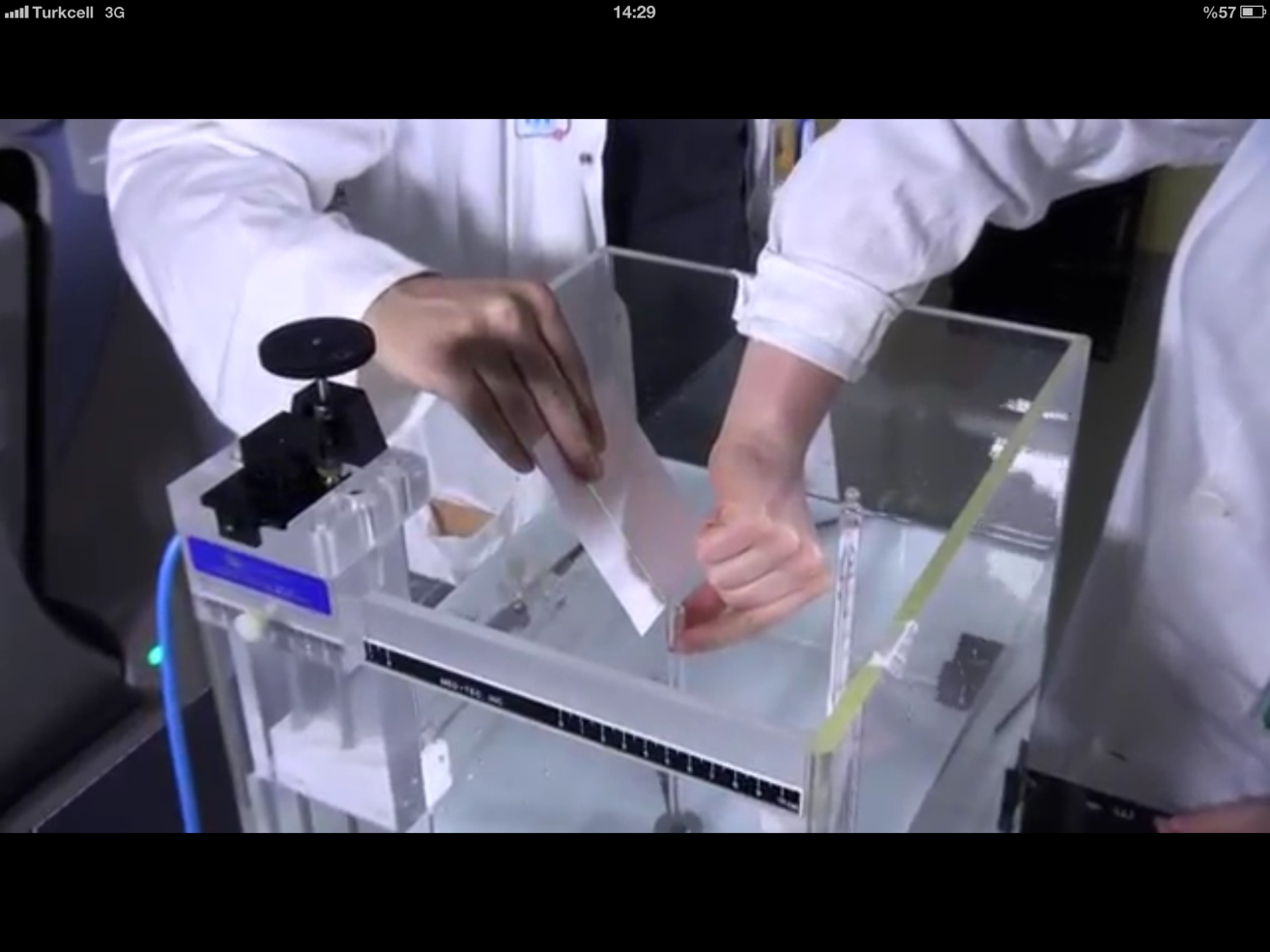


- 1. Add water to the container until it reaches the top level of the stick.


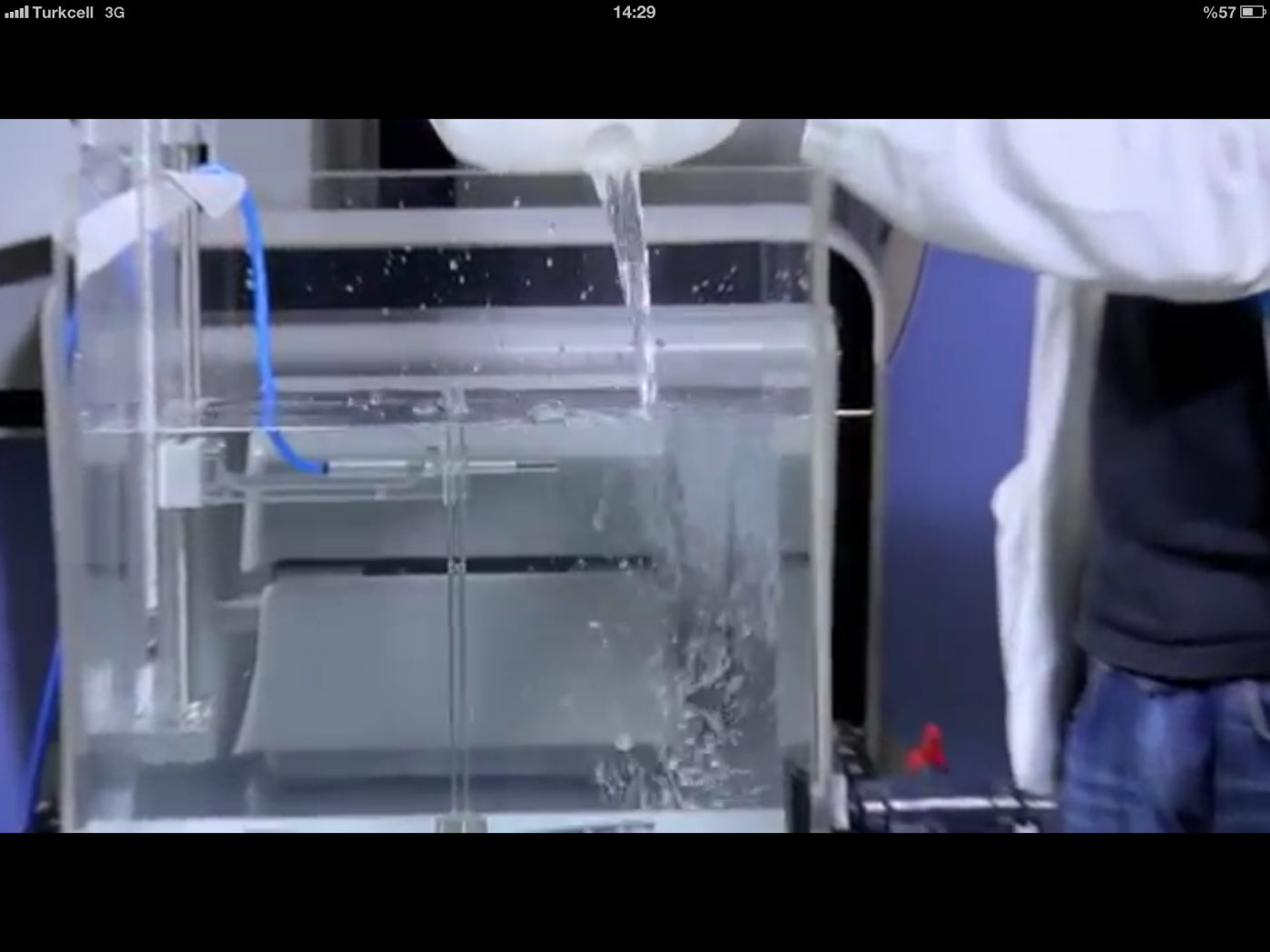


- 1. Adjust the treatment couch vertically so that the SSD is 100 cm at the water surface level.


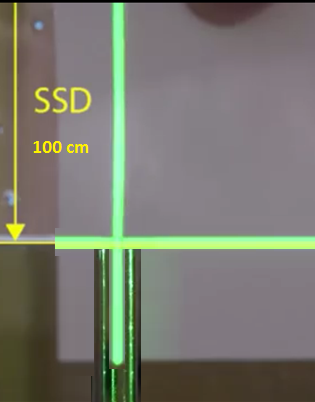


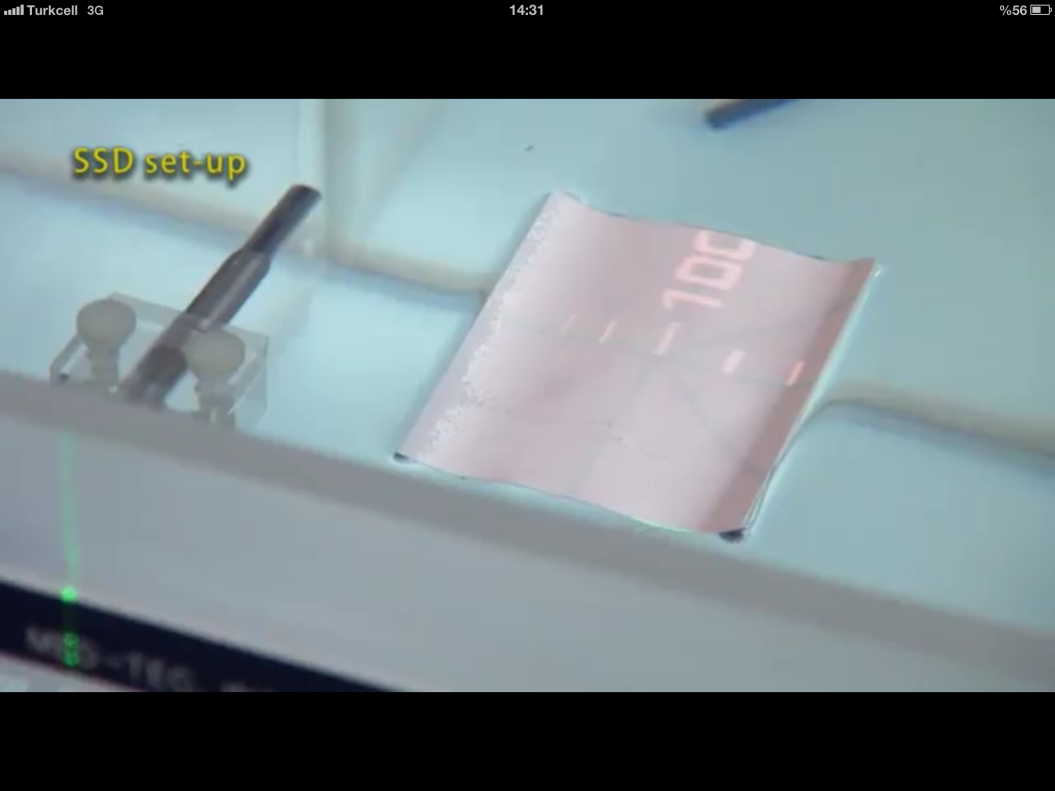


- 1. Using your TPS-calculated treatment time (MU), irradiate the first TLD individually with a 10×10 cm² field size, SSD of 100 cm at the water surface, to deliver 200 cGy to the TLD capsule placed at 10 cm depth.


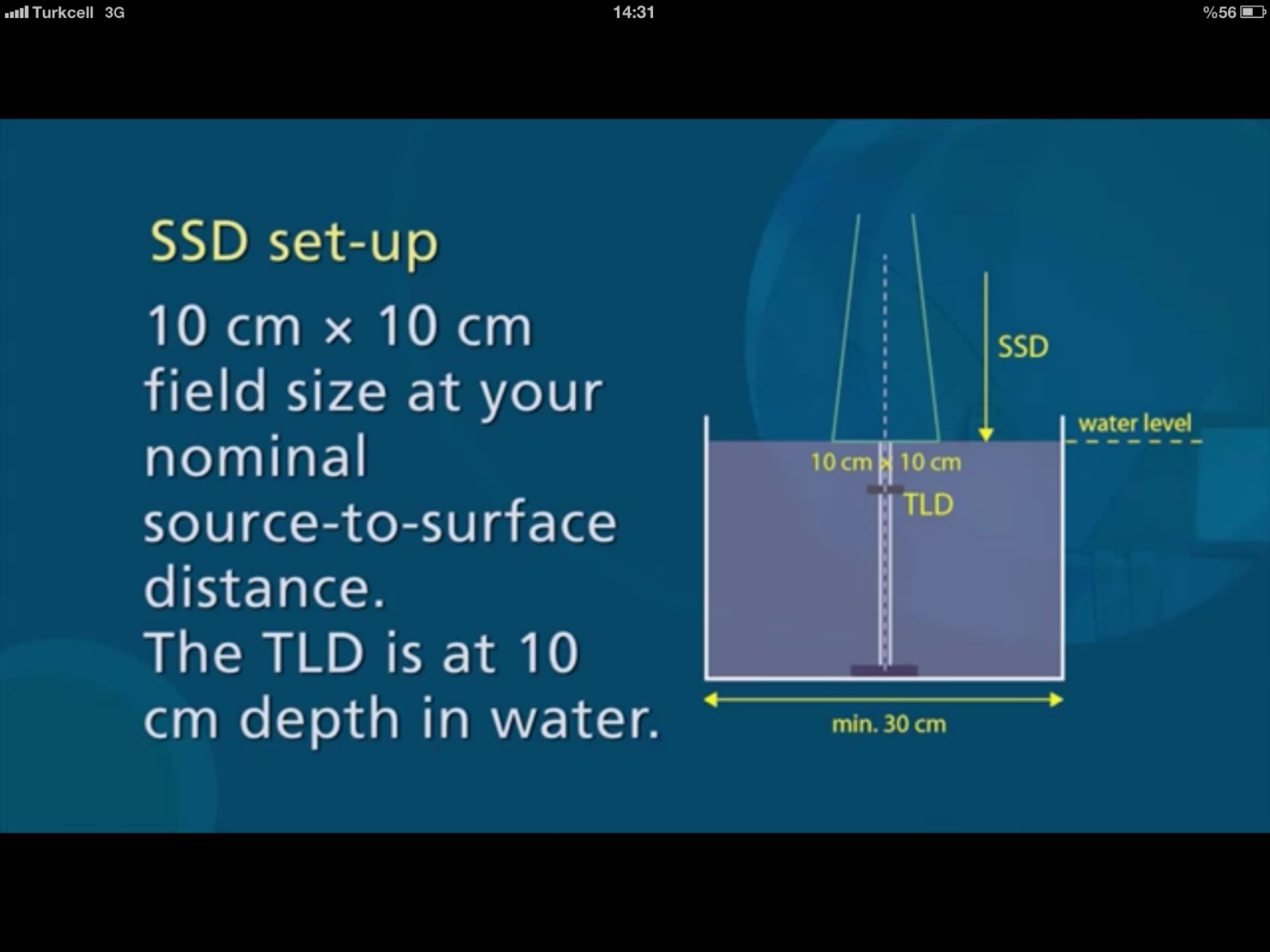


- 1. Remove the stick from the water and detach the first irradiated TLD from the stick. Place the second TLD into the cavity on the stick, aligning the green guide lines on the stick and the TLD capsule. Reinsert the stick into the water container slowly enough to prevent the TLD capsule from rotating, ensuring that it is centered along the beam’s central axis. **(Do not leave the first irradiated TLD inside the treatment room. Please make sure it is not exposed to any additional radiation.)**
  2. Using the same setup as for the first TLD, irradiate the second TLD with a 10×10 cm² field size, SSD of 100 cm, and 10 cm depth, using the treatment time calculated in your TPS to deliver 200 cGy.

1.
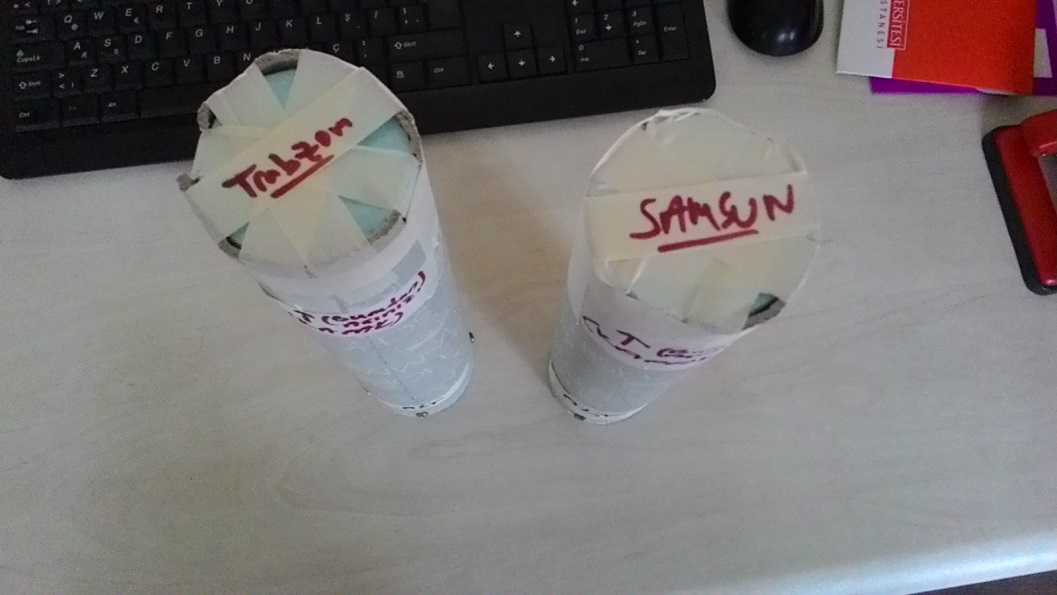
Please place the 1 control TLD (which was not irradiated), the 2 irradiated measurement TLDs, the completed and signed participation form, and the irradiation information form into the cylindrical protective container provided. Ensure the container is packaged securely to avoid damage during transport, and return it by courier with shipping costs paid on delivery.
