# Supplementary material for: Assessment of dose reliability in radiotherapy practices in Türkiye: A multicenter study
Source: J Appl Clin Med Phys. 2025 Aug 31;26(9):e70204. doi: 10.1002/acm2.70204 (PMC12398952; doi:10.1002/acm2.70204)
Supplement: Supplementary file 1 — Supporting Information [file ACM2-26-e70204-s001.zip › Appendix A - Aplication Form.docx]

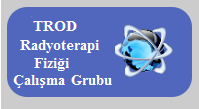
 - Turkish Society For Radiation Oncology – Radiotherapy Physics Working Group -

“**Assessment of Dose Reliability in Radiotherapy Practices in Türkiye: A Multicenter Study”**

| Aplication Participant Form |  | | |  |
| --- | --- | --- | --- | --- |
| Institution Name | |  | | |
| Institutional Study Code | |  | | |
| Responsible Radiation Oncologist | |  | | |
| Responsible Medical Physicist | | **Name and Surname** |  | |
|  |  | **Professional Experience (years)** |  | |
|  |  | **MSc Degree: YES / NO** |  | |
|  |  | **If Yes, Institution** |  | |
| City | |  | | |
| Institution Type (Private / Public / University) | |  | | |
| Mailing Address | |  | | |
| Email | |  | | |
| Phone Number | |  | | |
| TROD Working Group Contact | | **On behalf of the Working Group:** PhD.Med.Phys. XXXXXX (XXX University)  **Phone:** +XXXXXXX  **Email:** [XXXXXXXXXXXXX](mailto:murat.koylu@ege.edu.tr)  **Address:** XXXXXXXXXXXX | | |

**Study Guidelines**

1. This project is conducted by the TROD Radiotherapy Physics Working Group on behalf of TROD and consists of three phases.
2. Phase 1: TLD capsules will be sent to all centers and a 2 Gy irradiation will be requested.
3. Phase 2: Based on the Phase 1 results, free calibration training courses will be organized in regions found to require support.
4. Phase 3: TLD capsules will be sent again to all centers, and a second round of 2 Gy irradiations will be requested.
5. Institutions agreeing to participate are expected to complete all three phases.
6. Türkiye is divided into six (6) geographical regions for this study. Data will be reported anonymously, with no reference to institution name or city; only region and institution type will be disclosed.
7. Each participating center will be assigned a confidential study code, and dose results will remain confidential.
8. Unless specifically requested by the center, results will not be disclosed — even to the participating institution.
9. From each region, two (2) university hospitals, two (2) public hospitals, and two (2) private hospitals will be included (total 6 centers per region).
10. Participation is free of charge (no shipping or processing fees will be requested).
11. Centers wishing to participate voluntarily must complete and sign this form, and send it both with the TLD package and via email.

**Date:**

By signing this form, we voluntarily agree to participate in the study titled “Assessment of Dose Reliability in Radiotherapy Practices in Türkiye: A Multicenter Study” conducted by the TROD Radiotherapy Physics Working Group.

**Responsible Radiation Oncologist** **Responsible Medical Physicist**
