# Supplementary material for: Assessment of dose reliability in radiotherapy practices in Türkiye: A multicenter study
Source: J Appl Clin Med Phys. 2025 Aug 31;26(9):e70204. doi: 10.1002/acm2.70204 (PMC12398952; doi:10.1002/acm2.70204)
Supplement: Supplementary file 1 — Supporting Information [file ACM2-26-e70204-s001.zip › Appendix B - Irradiation Information Form.docx]

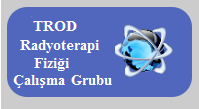
 - Turkish Society For Radiation Oncology – Radiotherapy Physics Working Group -

“**Assessment of Dose Reliability in Radiotherapy Practices in Türkiye: A Multicenter Study”**

| Irradiation Information Form |  | | |  | |  |
| --- | --- | --- | --- | --- | --- | --- |
| Institution Name/City |  | | | | |  |
| Institutional Study Code (leave this section blank) |  | | | | |  |
| Responsible Physicist |  | | | | |  |
| Calibration Protocol (398) |  | | | | |  |
| Irradiation Date |  | | | | |  |
| Brand/Model of Linac Used |  | | | | |  |
| Energy Used for Irradiation |  | | | | |  |
| Irradiation Depth | **10 cm** | | | | |  |
| Irradiation Field Shape | **10*10 cm^2^** | | | | |  |
| SSD | **100 cm** | | | | |  |
| Irradiation Technique | **SSD** | | | | |  |
| Irradiation Dose Amount | **200 cGy** | | | | |  |
| Irradiation Time (Mu) |  | | | | |  |
| Email |  | | | | |  |
| Phone |  | | | | |  |
| TROD Working Group Contact | **On behalf of the Working Group:** PhD.Med.Phys. XXXXX (XXXX University)  **Phone:** XXXXXXXXXXXX  **Email:** [XXXXXXXXXXXX](mailto:murat.koylu@ege.edu.tr)  **Address:** XXXXXXX | | | | |  |
|  |  |  |  | |  | |

**Note:** Please complete this form and send it via email.
